# Tracking the genetic diversity of SARS-CoV-2 variants in Nicaragua throughout the COVID-19 pandemic

**DOI:** 10.1038/s41598-024-84113-9

**Published:** 2025-02-09

**Authors:** Gerald Vásquez Alemán, Cristhiam Cerpas, Jose G. Juarez, Hanny Moreira, Sonia Arguello, Josefina Coloma, Eva Harris, Aubree Gordon, Shannon N. Bennett, Ángel Balmaseda

**Affiliations:** 1 Sustainable Sciences Institute, Managua, Nicaragua; 2 Laboratorio Nacional de Virología, Centro Nacional de Diagnóstico y Referencia, Ministerio de Salud, Managua, Nicaragua; 3 Division of Infectious Diseases and Vaccinology, School of Public Health, University of California, Berkeley, Berkeley, CA USA; 4 Department of Epidemiology, School of Public Health, University of Michigan, Ann Arbor, MI USA; 5 California Academy of Sciences, San Francisco, CA USA

**Keywords:** Public health, Epidemiology, Infectious diseases

## Abstract

The global circulation of SARS-CoV-2 has been extensively documented; however, the dynamics within Central America, particularly Nicaragua, remain underexplored. This study characterizes the genomic diversity of SARS-CoV-2 in Nicaragua from March 2020 through December 2022, utilizing 1064 genomes obtained via next-generation sequencing. These sequences were selected nationwide and analyzed for variant classification, lineage predominance, and phylogenetic diversity. We employed both Illumina and Oxford Nanopore Technologies for all sequencing procedures. Results indicated a temporal and spatial shift in dominant lineages, initially from B.1 and A.2 in early 2020 to various Omicron subvariants toward the study’s end. Significant lineage shifts correlated with changes in COVID-19 positivity rates, underscoring the epidemiological impact of variant dissemination. Comparative analysis with regional data underscored the low diversity of circulating lineages in Nicaragua and their delayed introduction compared to other countries in the Central American region. The study also linked specific viral mutations with hospitalization rates, emphasizing the clinical relevance of genomic surveillance. This research advances the understanding of SARS-CoV-2 evolution in Nicaragua and provides valuable information regarding its genetic diversity for public health officials in Central America. We highlight the critical role of ongoing genomic surveillance in identifying emergent lineages and informing public health strategies.

**Supplementary Information:**

The online version contains supplementary material available at 10.1038/s41598-024-84113-9.

## Introduction

Severe Acute Respiratory Syndrome Coronavirus 2 (SARS-CoV-2) caused the recent global pandemic of Coronavirus Disease 2019 (COVID-19)^1^. As of January 2023, 67 million cases have been reported worldwide, with the death toll surpassing 6.8 million^2^. Due to the novelty and severity of the virus, a complete genome was rapidly generated by January 2020 and published in February 2020^3^. The publication of this genome enabled the development of vaccines and the subsequent global vaccination effort. While COVID-19 disease severity has been greatly mitigated, SARS-CoV-2 continues to circulate, and its evolutionary dynamics include mutations associated with improved replication, increased virulence, and/or changes in the clinical progression of patient^4–6^^.^

New strategies for genomic surveillance through virus RNA sequencing have been implemented globally to meet this ongoing challenge^7^, making genomic surveillance accessible and indispensable for epidemiological research to understand the impact of viral evolution on SARS-CoV-2 transmission dynamics and COVID-19 disease.

Since its emergence, SARS-CoV-2 has continued to evolve into a variety of lineages, often with different properties, that may or may not persist^8,9^. The dissemination of sequencing technologies has been critical for tracking these genetic changes and the emergence of new lineages. Indeed, in response to the COVID-19 pandemic, many countries have invested in genomic sequencing as a component of health care infrastructure to track specific lineages or variants of concern and gain a deeper understanding of the pandemic’s dynamics locally. For example, the introduction of new genetic variants into a population has been associated with new waves of infection^10^. Variants differ in terms of transmission dynamics, disease severity, and mortality risk to infected individuals^11^. Finally, the introduction of new lineages has been associated with changes in risk of infection and disease severity in different age groups^12,13^ .

In Nicaragua, the COVID-19 pandemic began in mid-March 2020. Since then, several published studies have investigated the immunological impact of SARS-CoV-2 infection in various Nicaraguan cohorts^14–16^. However, the origin, diversity and dynamics of circulating lineages in Nicaragua have not been well-studied. Here, we document nationwide transmission dynamics of SARS-CoV-2 lineages. As such, this research also contributes insights into the evolution of SARS-CoV-2 in Central America, where there little information exists regarding genomic sequences due to limited infrastructure and low investment in genomic surveillance.

## Results

### SARS-CoV-2 genomes and associated data

Through epidemiological surveillance by the Ministry of Health in Nicaragua, nasopharyngeal swabs positive for SARS-CoV-2 via qRT-PCR with a Ct range of 18–30 were randomly selected for sequencing. A total of 1064 SARS-CoV-2 genomes with coverage exceeding 60% from all departments and autonomous regions of Nicaragua were recovered using Next Generation Sequencing with Illumina technology or Oxford Nanopore Technology (ONT) (Supplementary Fig. 1c) and were included in this study. Of the genomes analyzed, 1062 (99.8%) had patient sex data, with 620 (58.3%) being female and 442 (41.5%) being male. Additionally, hospitalization data were retrieved for 639 (60.1%) sequences (Supplementary Table 1). Over 40% of the recovered genomes originated from the capital of the country, Managua, where the National Virology Laboratory is located, followed by departments in the central region, Estelí and Matagalpa (7.89% and 7.33% respectively), and then the department of León (7.05%) on Nicaragua’s Pacific coast (Fig. 2 and Supplementary Table 3).

To compare the circulation of variants in Nicaragua to those circulating in the Central American region, we accessed publicly available sequences on the Global Initiative on Sharing All Influenza Data platform, GISAID (https://gisaid.org/) from 2020 to 2022, for a total of 22,530 genomes circulating in Guatemala (*n* = 4,255), Belize (*n* = 222), El Salvador (*n* = 863), Costa Rica (*n* = 9,210), Honduras (*n* = 332), and Panama (*n* = 6,584) (Supplementary Fig. 3).

## Phylogenetic diversity and epidemiological insights

SARS-CoV-2 sequences were assigned to clades using both the Nextstrain platform and nomenclature (https://clades.nextstrain.org/), as well as the Pangolin system and nomenclature (https://pangolin.cog-uk.io/). Nextstrain was able to classify all genomes successfully. However, Pangolin failed to classify 20 genomes, most of which belonged to the Omicron lineage.

**Fig. 1:**
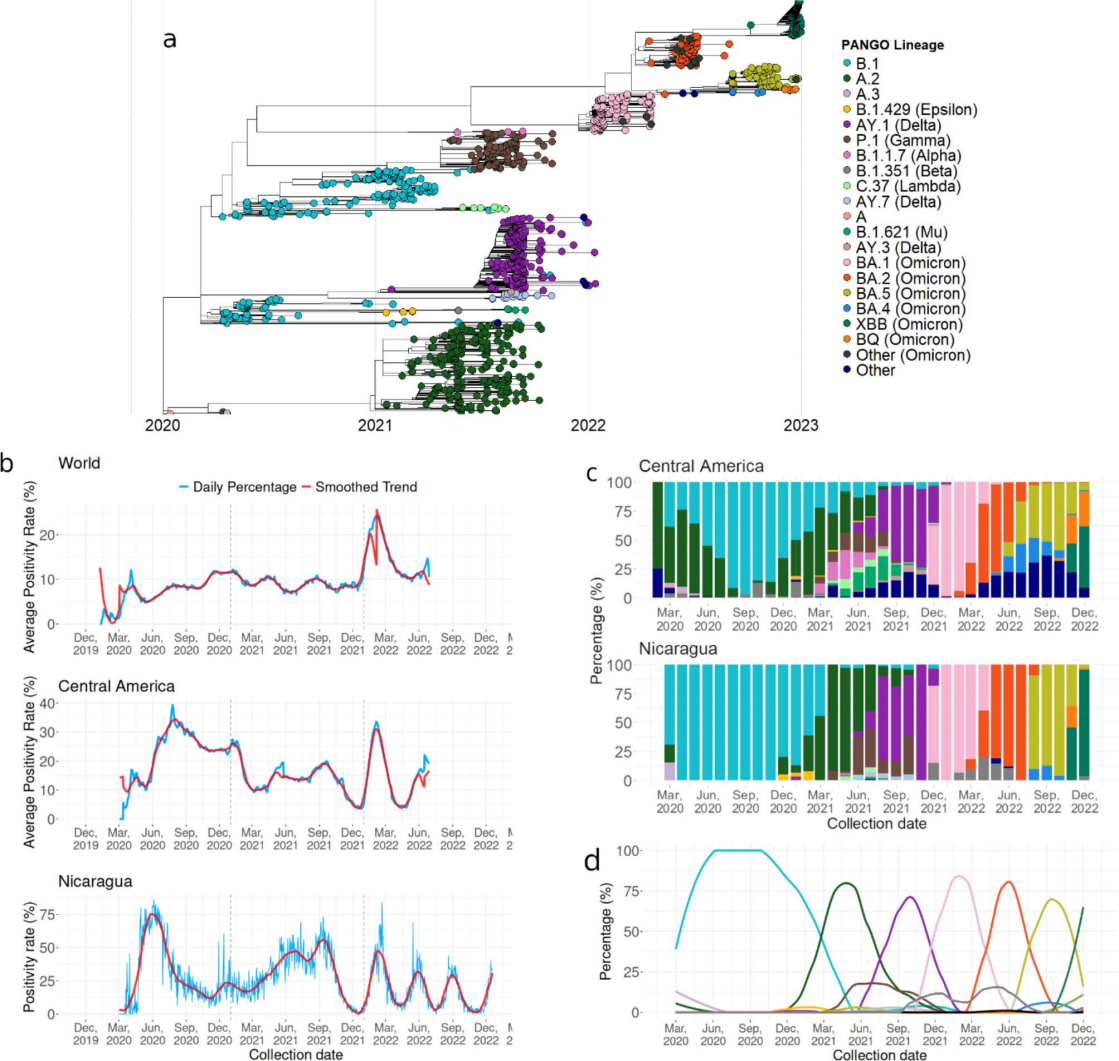
Diversity and temporal dynamics of SARS-CoV-2 lineages in Nicaragua. Lineages are color-coded. Legends present their Pangolin nomenclature, and their WHO Greek-letter designation if they have one based on their status as variants of concern. (a). Dated maximum likelihood tree of SARS-CoV-2 sequences from Nicaragua, tips colored according to the Pangolin nomenclature. (b) Positivity rate (blue line) and smoothed trend (red line) showing changes in COVID-19 positivity rates in Nicaragua, Central American countries, and the rest of the world from March 2020 to December 2022. The panel for Nicaragua expresses the daily positivity rate, while for Central America and the rest of the world, the average bi-weekly positivity rate was plotted. (c) Proportion of different SARS-CoV-2 lineages over time in Nicaragua and Central America, where colors correspond to Pangolin lineage, and height indicates the relative frequency of that lineage at a given time. (d) Monthly proportional trends of lineages circulating in Nicaragua, with colors representing different variants (corresponding to Pangolin lineages) and height indicating their relative frequency over time.

We used phylogenetic analysis to characterize the genetic diversity circulating in Nicaragua over the study period. Circulating lineages included variants of concern (VOC), as classified by the WHO, such as Alpha, Beta, Gamma, Delta, and Omicron. As in the global arena, the circulation of lineages in Nicaragua changed over time. The initial circulation of SARS-CoV-2 early in 2020 included Pangolin lineages A.1, A.2 and B.1; B.1 then dominated Nicaragua’s epidemiological landscape throughout 2021 and had the longest presence in the country (Fig. 1a and b). The A.2 lineage circulated from late 2020 to October 2021, simultaneously with B.1, making it the second longest circulating lineage. Although the AY.3 (Delta) lineage was identified by the end of 2020, it was not until mid-2021 that significant circulation of this lineage occurred, dominating the second half of 2021. The AY.1 and AY.2 lineages circulated infrequently. The BA.1 lineage, classified by WHO as Omicron, was identified in December 2021 and by January 2022 had completely replaced the previously circulating AY.3 lineage. Despite the rapid expansion of the BA.1 lineage, it did not circulate as long as previous lineages: after three months, the BA.2 lineage was introduced and became the dominant lineage in the country. During the circulation of BA.2, BA.4 was introduced but did not dominate as its predecessors did. In August 2022, the BA.5 lineage was identified and quickly replaced the circulation of the BA.2 and BA.4 lineages, circulating until November 2022, when the BQ and XBB lineages were introduced. By December 2022, the XBB lineage became the dominant lineage in the country. These results, shown in Fig. 1a and c, can be summarized as the dominance of a sole lineage for a long period of time in the early days of the pandemic, followed by more rapid turnover in the latter periods, including the co-circulation of multiple lineages for shorter periods until a new lineage was introduced.

**Fig. 2:**
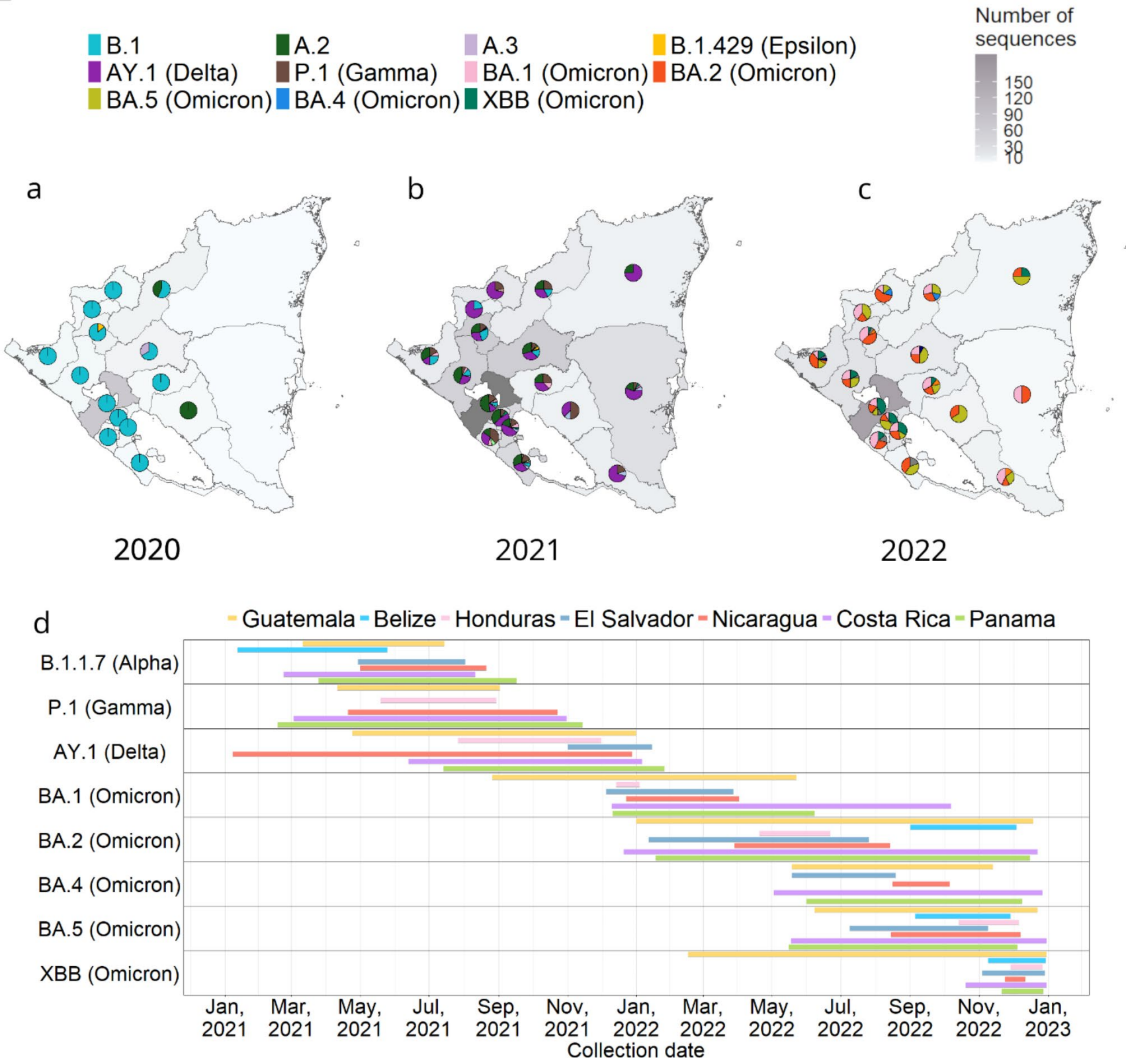
Spatial and temporal distribution of SARS-CoV-2 lineages. Frequency of genomes included in this study and distribution of lineages by department during the years 2020 (A), 2021 (B), and 2022 (C); in all three panels, the number of sequences per region is indicated by gray shadingth. (D) Temporal circulation of variants in Central American countries. Lineages are named using Pangolin nomenclature and their WHO Greek-letter designation if they have one.

In addition to different lineages circulating over time, we observed increases in the positivity rate, or percentage of cases testing positive, coinciding with the introduction of new lineages into the country. The first positivity peak after the initial introduction of SARS-CoV-2 into Nicaragua occurred in June 2020, coinciding with the fixation of the B.1 lineage (Fig. 1c). In November 2020, another spike in positivity rate corresponded with a resurgence of the A.2 lineage. In 2021, the positivity rate slowly increased, peaking in July 2021 when the A.2 and P.1 (Gamma) lineages co-dominated the epidemiological landscape, and again when the AY.3 lineage predominated in the second half of 2021. In 2022, four peaks in positivity rate were observed, coinciding with the circulation of the BA.1, BA.2, BA.5, and XBB lineages, respectively, towards the end of the year.

To compare the positivity rates between Nicaragua, Central American countries, and the rest of the world, we accessed the “Our World in Data” project repository on GitHub [https://github.com/owid/covid-19-data], where we extracted information on positivity rates (positive tests divided by total tests, expressed as a percentage) from countries around the world. Overall, we observed that the positivity rate patterns were very similar between Nicaragua and the average positivity rate of other Central American countries. When compared to the global positivity rate, similar patterns in peak increases were noted. Although the positivity rate in Nicaragua follows the same patterns as in Central American countries, it is higher than the average positivity rate in neighboring countries.

To achieve a better understanding of the circulation of SARS-CoV-2 lineages in Nicaragua, a trend graph was constructed using a LOESS linear model approach to simulate the monthly percentage trends of each lineage. This approach demonstrates patterns of emergence and disappearance of variants, which coincide with peaks in positivity rates (Fig. 1d).

## Spatio-temporal dynamics of SARS-CoV-2

Nationally, SARS-CoV-2 was detected in all departments of the country. Following the sequencing of 1,064 genomes nationwide, their corresponding lineages were identified. In 2020, the B.1 lineage predominated, with some occurrences of the A.1 and A.2 lineages (Fig. 2a). There were no genomes from the Río San Juan department or the South Caribbean Coast autonomous region during this year. In 2021, various lineages circulated throughout the national territory, predominantly the Delta variant lineages in the Pacific region, with AY.3 being prevalent, along with B.1 lineages in some areas, A.2, and P.1 (Gamma) (Fig. 2b). Similarly, in the Central region and the Autonomous Caribbean regions, the AY.3 lineage was most frequent. During 2022, all Omicron variant lineages were widely distributed throughout the country, nearly in equal proportions (Fig. 2c). The department of Managua had the highest number of sequences, corresponding to more cases over the three years studied, followed by the northern departments. The pattern of proportions of the number of sequences across the departments of Nicaragua was similar over the three years.

An inspection of the earliest sample collection dates of viral genomes in this study in Nicaragua was also conducted (Supplementary Table 2). Following the introduction of SARS-COV-2 into the country in March 2020, the earliest viral genomes belonged to the B.1, A.1, and A.2 lineages in the departments of Jinotega, Chontales, and Matagalpa, respectively. Similarly, other variants such as B.1.429 (Epsilon), AY.1 (Delta), and P.1 (Gamma) were initially reported in Estelí, RACCN, and Estelí, respectively, at different times from December 2020 to April 2021. This pattern of early detection suggests a wide geographical spread of virus variants across the national territory during the initial phases of the outbreak.

To characterize the circulation of variants in the Central American region by date, 22,530 genomes were downloaded from GISAID. Results indicate that many of the SARS-CoV-2 lineages circulating in Central America arrived early in their global spread (Fig. 2d). Belize, Costa Rica, Guatemala, and Panama show the earliest records of B.1.1.7 (Alpha) in the region. Nicaragua, although late to record most of the variants, stands out for the early introduction of the AY.1 (Delta) lineage followed by an extensive period of its circulation. Guatemala stands out as the earliest country in the region to record several variants and the first to record multiple Omicron variants, including BA.1 and XBB. Several of the variants, and XBB in particular, persisted in Guatemala for longer periods than in other countries, suggesting Guatemala may have been a critical entry point and dispersion hub for strains circulating in Central America. In general, our analysis emphasizes a sequence of introductions not confined to a single country but rather characterized as a mosaic of introductions across various points in Central America.

**Fig. 3:**
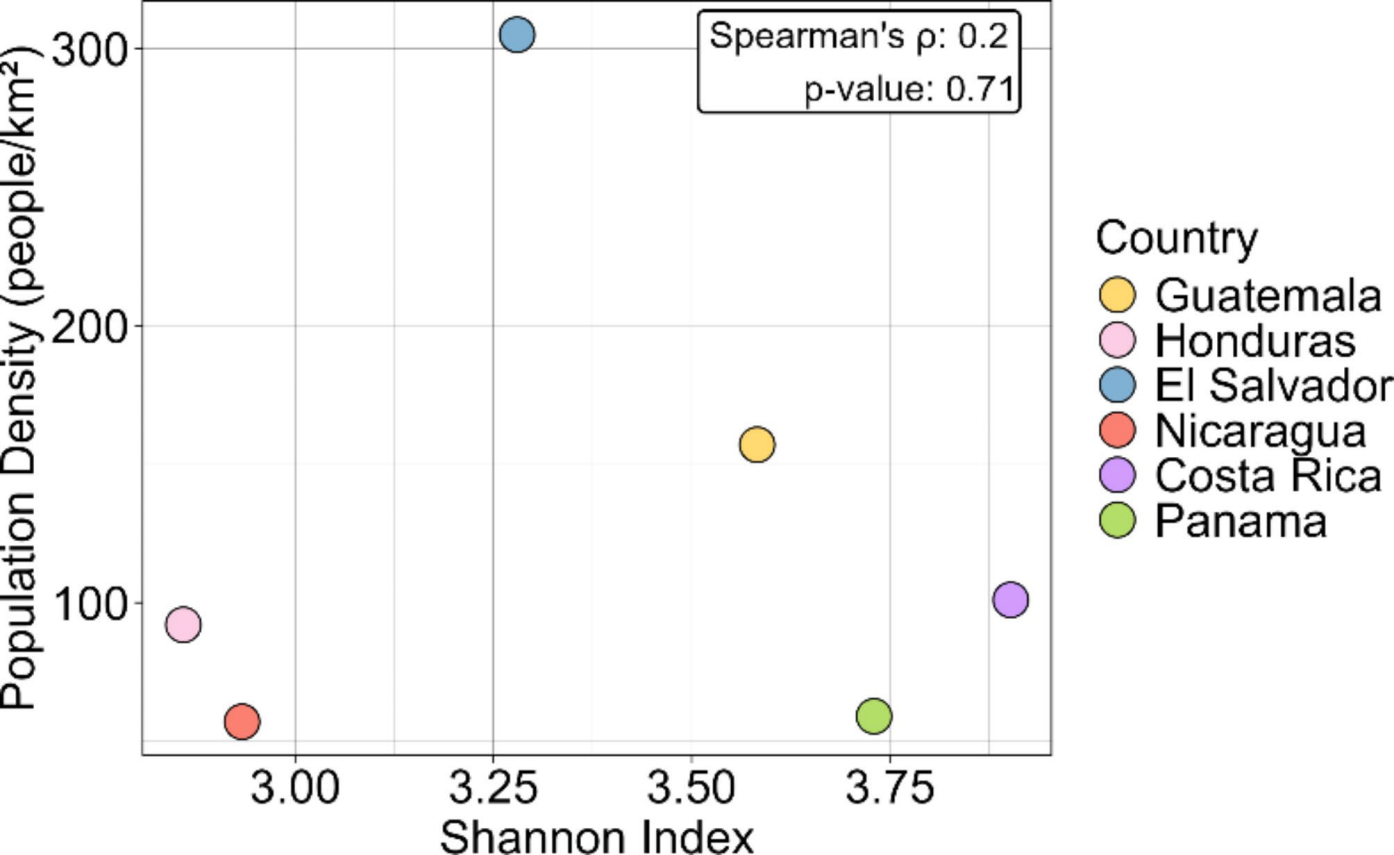
Diversity of circulating lineages in Central America: Shannon diversity index of SARS-CoV-2 lineages versus population density (people per square kilometer) in Central American countries.

To further examine how Central American countries have hosted a diversity of variants, we compared them based on the Shannon diversity indices of the lineages. Costa Rica and Panama hosted the highest diversity of lineages (3.90 and 3.73 respectively), followed by Guatemala (3.58) and El Salvador (3.27). Nicaragua and Honduras had the lowest diversity of lineages (2.93 and 2.85, respectively) (Supplementary Fig. 5). We plotted the diversity indices of countries against their population density (people per square kilometer) (Fig. 3). Although no statistically significant association was found between population density and the diversity index, it was observed that Nicaragua and Honduras, which have the lowest population densities, also exhibit the lowest diversity indices. El Salvador and Guatemala, with higher population densities, display higher diversity of lineages. However, Costa Rica, despite having a population density similar to that of Honduras, shows high diversity. Finally, it was observed that Panama, despite its low population density, also has a considerably high diversity of lineages. To determine whether there is a statistically significant correlation between population density and the population of Central American countries, a Spearman correlation test was conducted. The test yielded a p-value of 0.71 and a correlation coefficient of 0.2, indicating no statistically significant correlation.

**Fig. 4:**
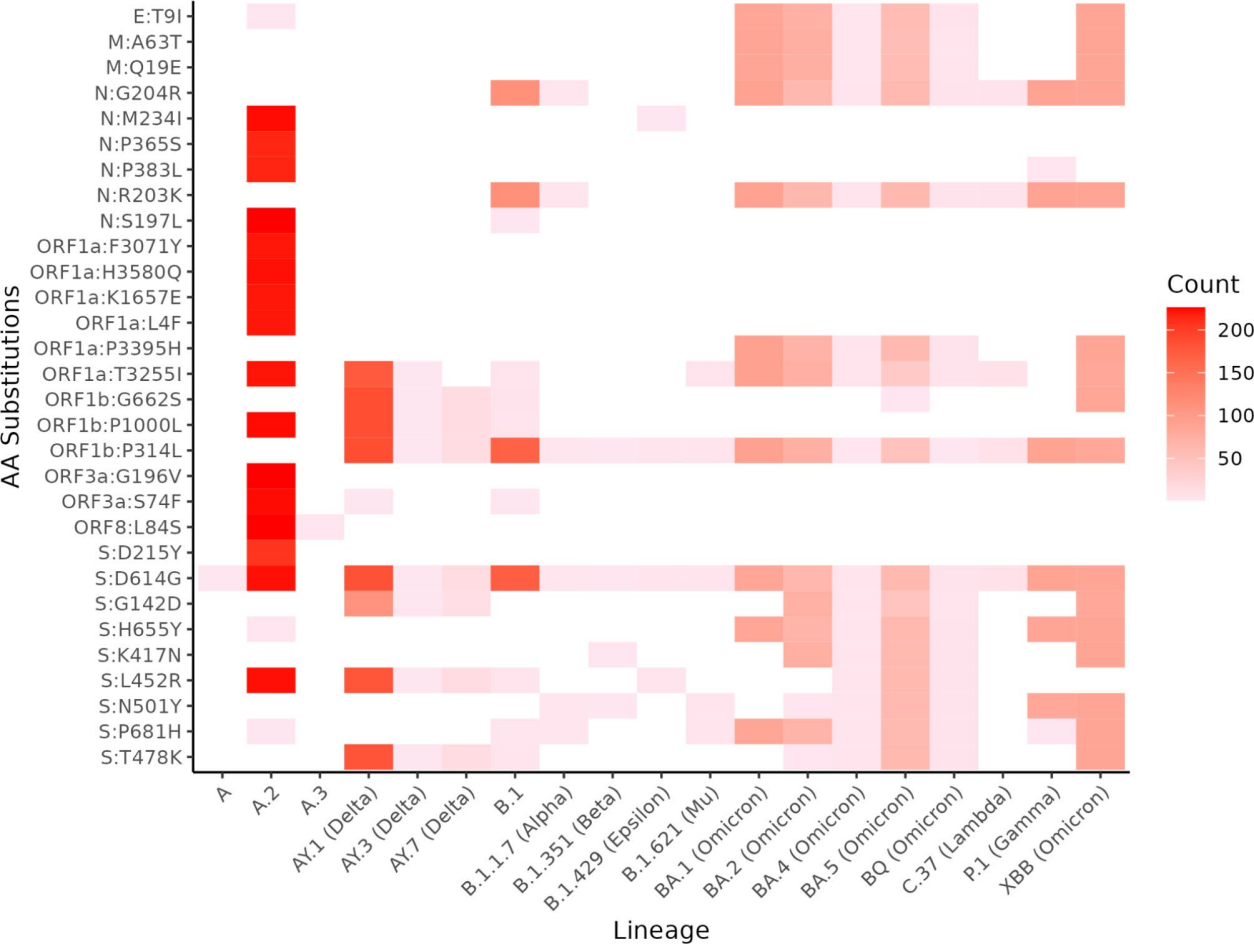
Number of mutations in lineages circulating in Nicaragua. Heatmap indicates the number of times a specific amino acid (aa) substitution (y-axis) was observed among our sequences within a given lineage (x-axis). Lineages are named using Pangolin nomenclature and their WHO Greek-letter designation if they have one. Substitution nomenclature follows standard practice: gene: original aa state, gene position, new aa state.

Given the extensive range of mutations observed in SARS-CoV-2 genomes, we assessed the top 30 most frequent amino acid substitutions occurring among the sequences from Nicaragua by lineage (Fig. 4). Within the A.2 lineage, which circulated extensively throughout much of 2021, a high number of the top 30 amino acid substitutions was identified. This lineage was followed by the Delta variant lineages and B.1, which both had high counts but across fewer of the top 30 substitutions. The Omicron lineages accumulated high counts, and notably, across most of the top 30 amino acid substitutions.

The substitution in the spike gene from aspartic acid (D) to glycine (G) at gene position 614 (S: D614G) was present in all genomes except for lineage A.3. Following this, the P314L mutation in the ORF1b gene region was the second most prevalent substitution across the genomes, only absent in lineages A, A.2, and A.3.

**Fig. 5:**
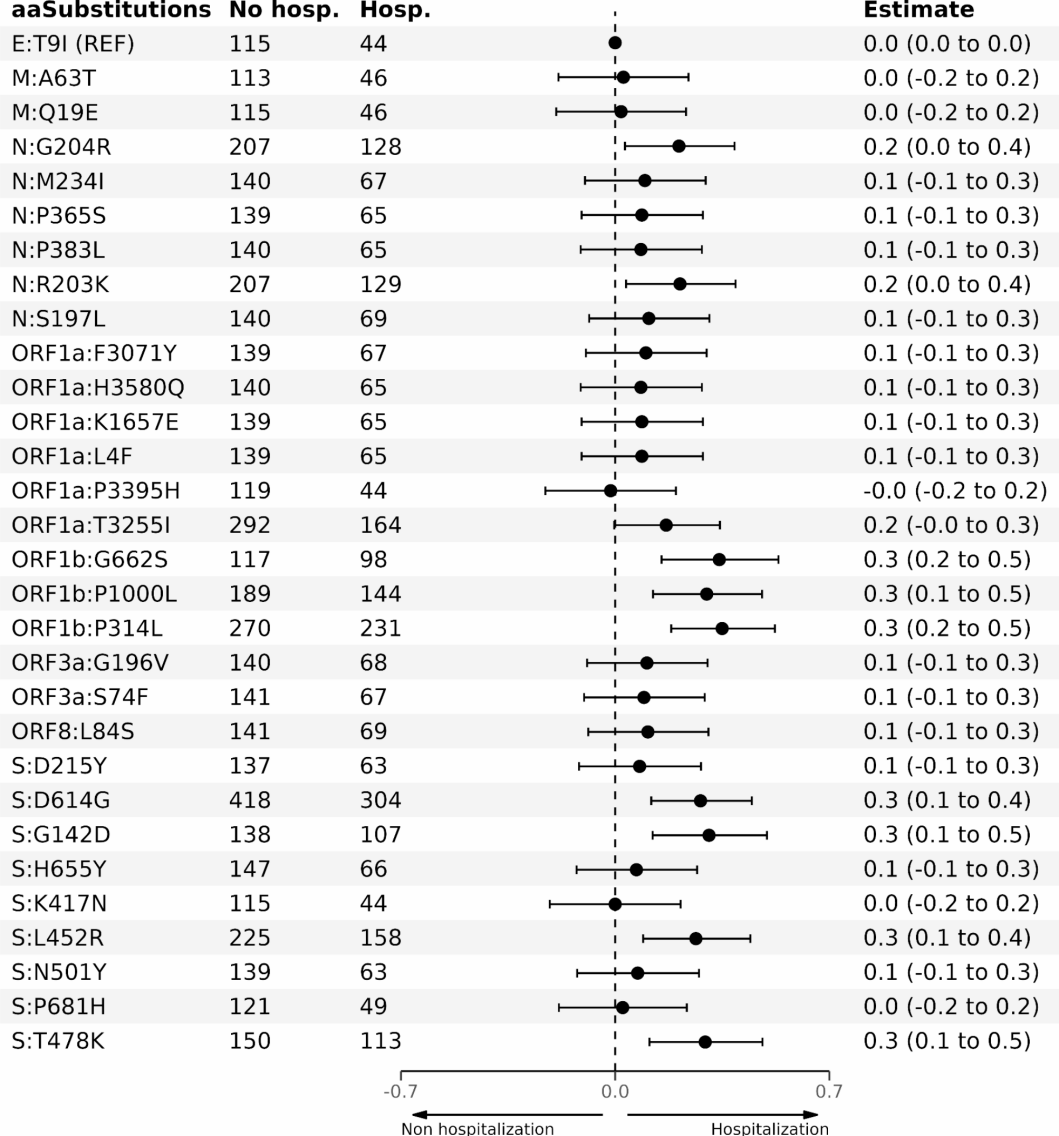
Logistic regression model with the 30 most frequent mutations in SARS-CoV-2 genomes against hospitalization outcome. The estimate is exponentiated and represents the Odds Ratios (ORs) on a log_10_ scale where 0 represents no change in risk, values above 0 indicate increased risk, and values below 0 indicate decreased risk of hospitalization.

We analyzed the association between the top 30 mutations and the likelihood of hospitalization (Fig. 5), finding significant associations between hospitalization and specific mutations in the N region (G204R and R203K), ORF1b region (G662S, P1000L, P314L), and spike protein (D614G, G142D, L452R, and T478K).

## Discussion

This study represents the first characterization of the genomic diversity of SARS-CoV-2 lineages circulating in Nicaragua during the first three years since the initial COVID-19 case in March 2020 and is the result of a multi-center collaboration to establish genomic epidemiological surveillance within the country. Further, many studies often overlook the Central America region, highlighting the critical need for research in this area. Our study helps fill this gap by presenting the diversity of SARS-CoV-2 lineages that circulated not only in Nicaragua but across Central America during the first three waves of the COVID-19 pandemic.

During the initial phase of the study, when samples were sent abroad for sequencing, there was no strict stratification by department in the sample selection. However, this stratification approach was implemented in the second phase once local sequencing was established. A strength of this study is the integration of diagnostic processes for epidemiological surveillance with sequencing for genomic surveillance, which are co-located and interconnected. This setup allowed for near-immediate access to samples, the ability to perform sequencing in real-time, and the strategic selection of samples based on the geographic origin of the positive cases.

Lineage classification was performed using the Nextstrain platform, with 1.88% of the genomes not successfully classified. While there is no consensus on the methodology for viral lineage classification, most approaches, including those of Nextstrain and PANGO, aim to classify viral genomes based on their affiliation with a phylogenetic clade or cluster. However, some viral genomes do not neatly cluster within a single group, especially in the Pangolin system, which includes mutations and single nucleotide polymorphisms in the classification. In such cases, these genomes are classified as “recombinants” or “unclassified”^17^. We found that that genomes labeled as “unclassified” were predominantly from the period of Omicron circulation, which underwent high diversification due to the variants’ rapid transmission capabilities^13,18^.

Much like the global trajectory of the pandemic, our data demonstrate patterns of dominance and replacement among different virus lineages over time. During the first year and a half of the pandemic, the B.1 and A.2 lineages were dominant, even as new lineages were emerging in different parts of the world, particularly in European countries, the United States, and Brazil^17,19^. These patterns are similar to those observed in many countries on the African continent, where the circulation of B.1 sublineages was predominant during the first year of the pandemic^20,21^. The dynamics in Nicaragua shifted in 2021 with the rapid emergence and transition between lineages, such as the replacement of Delta (AY.3) by Omicron (BA.1). In 2022, multiple Omicron lineages circulated, leading to notable changes in positivity patterns.

At a regional level, Nicaragua is part of a multi-country network of viral exchange involving many of its closest neighbors, including Guatemala, Honduras, and Costa Rica. There is a noticeable disparity in the number of sequences available across Central American countries. For instance, Belize, El Salvador, and Honduras have reported a very low number of sequences, and these are limited to specific periods. In contrast, Panama and Costa Rica have a significantly higher number of sequences. While Nicaragua does not have as many sequences as its southern neighbors (Costa Rica and Panama), it has a consistent distribution of sequences over the duration of this study. Although we were unable to demonstrate a direct link between a country’s population density and the diversity of viral lineages, certain patterns emerged. Countries with low lineage diversity, such as Honduras and Nicaragua, also tend to have low population densities, while those with high population densities, like Guatemala and El Salvador, exhibit greater lineage diversity. Previous studies have suggested that population dynamics may significantly influence the pandemic’s impact, particularly in terms of morbidity and mortality^22,26^.

Our findings align with the study by Okoy et al.^27^, which analyzed variant diversity across continents. This report showed that Europe, followed by North America, had the highest diversity of circulating variants, with Asia trailing behind. In contrast, South America, Africa, and Oceania exhibited significantly lower diversity levels^27^. The authors suggest that these differences may be influenced by varying sequencing capacities across regions as well as the dynamics of travel, international exchanges, and population movements between cities.

According to the data analyzed in this study, the diversity of lineages circulating in Nicaragua was lower compared to most Central American countries, and the introduction of new lineages occurred later than in much of the region. It has been documented that most Central American countries, except for Nicaragua, implemented stringent lockdowns and states of emergency^28,33^. Thus, beyond these public health measures, other intrinsic factors in population dynamics may influence the diversity and establishment of SARS-CoV-2 lineages.

At the Central American level, despite differences in the diversity and timing of lineage introductions between Nicaragua and the rest of Central America, it is notable that, overall, similar patterns of variant introductions, disappearances, and positivity peaks were observed. However, Nicaragua consistently exhibited a higher positivity rate compared to the Central American average. This could be attributed to the higher infection rate in the country; as mentioned earlier, Nicaragua did not implement strict lockdown measures like other Central American nations. Additionally, the sampling systems may influence the positivity rate. Nicaragua’s healthcare surveillance system, being a public, community-based, and interconnected system, could capture suspected positive suspected cases, contributing to the higher positivity rate.

We identified a relationship between SARS-CoV-2 genomic mutations and hospitalization. The mutations identified in the spike protein, particularly D614G, which have been associated with hospitalization, are consistent with previous findings linking virus transmissibility to disease severity^34,36^. Due to its prevalence and association with increased infectivity, this mutation could indicate a more severe clinical course, resulting in a higher number of hospitalizations. In addition to spike mutations, our analysis identified a recurrence and association of mutations in the ORF1b region among hospitalized patients, suggesting that alterations in the viral replicative machinery may directly impact viral pathogenesis. The viral polymerase function, encoded by ORF1b, is critical for genome replication, and mutations enhancing this process could theoretically increase virulence. Overall, our data align with previous studies showing an association between hospitalization and mutations in specific regions of the SARS-CoV-2 genome^37,39^. However, the association between the S: D614G and ORF1b: P314L mutations and hospitalization rates, even in lineages like Omicron not typically linked to severe outcomes, reflects a complex interplay of viral evolution and host factors. These mutations might be part of an ongoing adaptation of the virus, where emerging lineages are evolving to become less virulent. Additionally, the impact of these mutations on disease severity could be significantly influenced by population immunity, whether from infection or vaccination, which could alter the clinical outcomes of infections with these variants. Furthermore, multifactorial influences such as access to healthcare, comorbidities, age, and individual immune responses play critical roles in determining disease severity. In our analysis, we have identified both single and shared mutations across different SARS-CoV-2 lineages. Mutations can emerge early in epidemics, providing advantages to subsequent generations of lineages, as seen with the D614G mutation^40^. We also observed the same mutations in different lineages, likely due to convergent evolution driven by similar selective pressures. This phenomenon, where different lineages develop the same mutation due to similar conditions despite not being genetically linked, has been documented^41,42^. This study provides a general overview of mutations in SARS-CoV-2 Nicaraguan sequences and aligns with findings from other research^34,39^. However, it does not have the scope to analyze selective pressures in detail. Additionally, a more in-depth analysis is necessary to thoroughly assess the impact of individual mutations and the interactions among sets of mutations on the risk of severe disease and hospitalization.

Genomic surveillance requires moderate to high viral loads to yield adequate sequence information. Samples reaching the National Virology Laboratory (NVL) in Managua for processing are also at risk of degradation of genetic material due to transportation time, which is dependent on travel distance; thus, samples from more distant locations may not achieve an appropriate cycle threshold (Ct) for sequencing, despite having positive PCR results. As a result, fewer sequences might be derived from regions farther from Managua. These regions are also typically less populated, resulting in fewer samples sent to the National Center for Disease Control and Prevention (CNDR) for sequencing. Despite these challenges, our study provides a comprehensive geographic representation of sequences across Nicaragua that supports our observation of sustained SARS-CoV-2 transmission and the nationwide distribution of lineages throughout the three years of this study. We acknowledge that the number of genomic sequences available from various countries depends on their capacity to perform genomic surveillance and sequencing, which may introduce bias into our results.

This study was made possible by national viral and clinical surveillance programs in Nicaragua coupled with recent investments in genomic sequencing and analytical capacities. Together, these initiatives have provided new insights into the dynamics of the SARS-CoV-2 pandemic in Nicaragua and the circulation of variants at the national and regional levels.

## Methods

### Study population and sampling collection

Respiratory swab samples were collected between March 2020 and December 2022 across 15 departments and 2 autonomous regions in Nicaragua, as part of two ongoing studies in Managua: the Nicaraguan Pediatric Influenza Cohort Study (NPICS)^14,43^and the Household Influenza Cohort Study (HICS)^15,44^, along with the Nicaraguan national surveillance of respiratory diseases. All the methods were performed in accordance with the relevant guidelines and regulations of the Nicaraguan Ministry of Health.

The NPICS is conducted in the catchment area of the “Sócrates Flores Vivas” Health Center (HCSFV), the principal primary care facility serving neighborhoods along the shores of Lake Xolotlán in Managua, and follows ~ 2781 children. The HICS follows ~ 436 households and is conducted in the same area as the NPICS and was extended to SARS-CoV-2 in March of 2020. Additionally, samples from all provinces of the country were collected through the National Surveillance Program of the Nicaraguan Ministry of Health. The National Epidemiological Surveillance System for SARS-CoV-2, implemented by the Ministry of Health, is a monitoring network whose main objective is to track COVID-19 cases across all regions of Nicaragua. This system employs quantitative Reverse Transcription Polymerase Chain Reaction (qRT-PCR) to diagnose SARS-CoV-2 cases, enabling tracking of viruses as they emerge. The Ministry of Health is organized into 19 Local Comprehensive Health Care Systems (SILAIS), which are responsible for monitoring COVID-19 cases in their respective localities, managing epidemiological surveillance from primary care centers and local hospitals. In this way, the National SILAIS Network functions as a decentralized structure, where each SILAIS is a contributes to the National Epidemiological Surveillance System for SARS-CoV-2, thus enabling monitoring and surveillance of the SARS-CoV-2 cases at the national level.

All SARS-CoV-2 positive samples identified by qRT-PCR were potential candidates for genomic sequencing. However, due to limited sequencing capabilities within the country during the initial phase of the pandemic, between March 2020 and July 2021, a subset of positive samples was randomly selected and sent to the Icahn School of Medicine at Mount Sinai in New York for whole-genome sequencing using Illumina technology. The resulting sequences were then sent back to Nicaragua for further analysis. During this period, the selection of the respiratory swabs was not stratified by department; instead, random selection was performed among all positive cases across the country.

As a response to the COVID-19 pandemic, genomic surveillance was implemented in Nicaragua starting from July 2021 with the Oxford Nanopore Technology (ONT) sequencing. As part of this initiative, respiratory swabs with Ct values ranging from 18 to 30 were randomly selected, stratified by department based on the number of positive COVID-19 cases reported in the national surveillance data provided by the Ministry of Health. While we aimed to maintain a rigorous stratified sampling process by department, we had no control over the sampling strategies employed by the national health surveillance, which could change over time depending on the epidemiological landscape in the country. Additionally, as is typical in sequencing studies, some samples yielded sequences of poor quality, which were excluded from the analysis. This approach ensured that the selection process was both randomized and reflective of the epidemiological distribution of SAR-CoV-2 across departments, albeit with the noted limitations due to external sampling methodologies and inherent challenges in sequencing quality. From all successfully sequenced samples, those with a coverage of 60% or higher (as a quality parameter) were chosen.

In total, 566 genomes were obtained from Illumina sequencing in the United States, and 498 genomes were obtained in Nicaragua (336 using the Artic V3 sequencing protocol and 162 using the Oxford Nanopore Technology Midnight protocol).

## RNA extraction and RT-PCR

Samples suspected of SARS-CoV-2 infection were collected during the acute phase of illness and processed at the National Virology Laboratory (NVL). Viral RNA was extracted using the QIAmp viral RNA mini kit (QIAGEN, Germany) following the manufacturer’s instructions. In brief, the viral particles in 140 µl of sample were lysed using the lysis buffer provided to release the viral RNA. The lysate was then combined with a binding buffer, which aids the attachment of viral RNA to the silica membrane in the spin column. The RNA bound to the membrane was thoroughly washed several times with 500 µl of wash buffers to eliminate impurities and other contaminants. The purified RNA was then eluted from the spin column using 60 µl of elution buffer.

Subsequently, qRT-PCR was performed following the standardized multiplex PCR protocol developed by the Virology Institute at Charité University Hospital (Corman et al., 2020), using the ABI 7500 Fast PCR platform (Applied Biosystems, Foster, CA, USA). Throughout the pre-analytical and analytical process, both the samples and viral RNA were handled in type II biosafety cabinets and with all required personal protective equipment.

## Library preparation and next generation sequencing

In Nicaragua, the ONT library was prepared according to the ARTIC V3 or ONT Midnight protocol for PCR tiling, using the rapid barcoding kits SQK-LSK109 or SQK-RBK110.96, respectively. Sequencing was conducted on the MinION MK1B and MinION MK1C platforms. First, viral RNA was reverse-transcribed using LunaScript RT SuperMix. Subsequently, the genetic material underwent PCR amplification aimed at amplifying almost the entire SARS-CoV-2 genome. For this step, both the ARTIC V3 and Midnight protocols used separate primer pools for overlapping tiled PCR reactions spanning the viral genome. The result was DNA segments ranging from 400 to 1200 base pairs covering the SARS-CoV-2 genome, which were confirmed by 1% agarose gel electrophoresis.

The library preparation process for the ARTIC V3 network protocol includes a DNA end-prep step and subsequent addition of barcodes, after which all individually barcoded samples were pooled into a single library. After a DNA purification step using magnetic beads, molecular adaptors for sequencing were added. In the ONT Midnight protocol, following amplicon generation, the segments were exposed to tagmentation enzymes that both cut the amplicons and add barcodes. Once the samples were barcoded, a single library was created, and the genetic material was purified using magnetic beads, 70% alcohol and molecular grade water.

AmpureXP purification beads (Beckman Coulter, High Wycombe, UK) were used to clean up the PCR products. The DNA concentration (PCR products and DNA libraries) was measured using the Qubit dsDNA HS Assay Kit for fluorometric DNA measurement (Thermo Fisher Scientific) on a Qubit 4.0 Fluorometer. The final DNA library was then loaded onto a primed MinION flow cell R9.4 (FLO-MIN 106).

### Consensus genomes

During sequencing, the ONT MinKnow software discarded all sequences with a Phred quality score below 8. The resulting fastq files were trimmed and mapped to a reference genome and then assembled to generate consensus sequences using the ARTIC network bioinformatic pipeline (https://artic.network/ncov-2019). For samples sequenced using the ONT Midnight protocol, the bioinformatics pipeline wf-artic was used, incorporated into the ONT EPI2ME platform.

### Data analysis and visualization

Descriptive and statistical analyses of the data were conducted using the R language, version 4.3.1, with the RStudio Integrated Development Environment version 2023.06.2. Data visualization was performed using the ggplot2 R package.

### Phylogenetics analysis

Lineage determination and amino acid substitution analysis were conducted using the Nextstrain platform. Sequence alignments were performed using MAFFT v7.4 and manually inspected with AliView v1.28. Construction of Maximum Likelihood (ML) phylogenetic trees was carried out using IQTREE version 2.2.2.7 with 1000 bootstrap replicates, employing the ModelFinder feature to select the best nucleotide substitution model. The software TreeTime was used to convert the raw ML tree into a dated tree^45^, which estimates evolutionary rates and the Time to the Most Recent Common Ancestor (Tmrca) via least-squares regression, without employing a preset clock rate by default. Instead, it estimates the rate using linear regression according to the data^46^. The root-to-tip regression estimated a rate of 1.125 × 10^−3substitutions/site/year, with an R² of 0.95 (Supplementary Fig. 4). It was decided to adopt this approach due to its lower demand on computational resources and its proven satisfactory results in previous studies^47^. The R package ggtree was employed for visualizing and annotating the dated ML tree.

## Electronic supplementary material

Below is the link to the electronic supplementary material.

Supplementary Material 1

## Data Availability

The genome sequences obtained in this study have been made available through the Global Initiative on Sharing All Influenza Data (GISAID) repository https://gisaid.org/. Accession numbers are provided in Supplementary Table 4.
